# Chemical biology tools to interrogate the roles of *O*-GlcNAc in immunity

**DOI:** 10.3389/fimmu.2022.1089824

**Published:** 2023-01-26

**Authors:** Abhijit Saha, Alberto Fernández-Tejada

**Affiliations:** ^1^ Chemical Immunology Lab, Centre for Cooperative Research in Biosciences, CIC bioGUNE, Basque Research and Technology Alliance (BRTA), Derio, Biscay, Spain; ^2^ Ikerbasque, Basque Foundation for Science, Bilbao, Spain

**Keywords:** *O*-GlcNAc, chemical immunology, chemical tools, immunity, glycobiology

## Abstract

The *O*-linked β-*N*-acetylglucosamine (*O*-GlcNAc) glycosylation of proteins is an essential and dynamic post-translational modification in mammalian cells that is regulated by the action of two enzymes. *O*-GlcNAc transferase (OGT) incorporates this monosaccharide on serine/threonine residues, whereas *O*-GlcNAcase (OGA) removes it. This modification is found on thousands of intracellular proteins involved in vital cellular processes, both under physiological and pathological conditions. Aberrant expression of *O*-GlcNAc has been implicated in diseases such as Alzheimer, diabetes, and cancer, and growing evidence over the last decade has also revealed key implications of *O*-GlcNAcylation in immunity. While some key signaling pathways involving *O*-GlcNAcylation in immune cells have been discovered, a complete mechanistic understanding of how *O*-GlcNAcylated proteins function in the immune system remains elusive, partly because of the difficulties in mapping and quantifying *O*-GlcNAc sites. In this minireview, we discuss recent progress on chemical biology tools and approaches to investigate the role of *O*-GlcNAcylation in immune cells, with the intention of encouraging further research and developments in chemical glycoimmunology that can advance our understanding of *O*-GlcNAc in immunity.

## Introduction

The β*-O*-linked glycosylation with *N*-acetylglucosamine (*O*-GlcNAcylation) is a dynamic post translational modification that plays an essential role in the regulation of cellular functions, such as cell cycle and metabolic processes in response to nutrient and stress stimuli (1, 2). Its dynamic behaviour is controlled by two opposing enzymes: *O*-GlcNAc transferase (OGT) installs this sugar moiety onto serine (Ser) and/or threonine (Thr) residues (3, 4) and *O*-GlcNAcase (OGA) hydrolyses it (2, 5). Early studies in mammals have found that genetic knockout of OGT is embryonically lethal, whereas knockout of OGA is perinatally lethal (6, 7). Accumulating evidence has shown the essential roles of these enzymes in maintaining cellular homeostasis, with their dysregulation being associated with numerous diseases. The human immune system works in a complex fashion with a finely tuned regulatory mechanism. The adaptive and innate immune responses are responsible for sensing, identifying, and eliminating potentially harmful and pathogenic substances. Although many studies have investigated the functions and mechanisms of *O*-GlcNAcylation in important cellular processes, the specific roles of *O*-GlcNAc in immunity have not been fully elucidated. Using modern chemical approaches, some recent preliminary studies performed in specific immune cells, especially T cells, have demonstrated the immunological significance of this modification. Thus, OGT was found to be essential for T cell activation (8) and Notch, the T cell antigen receptor (TCR) and the transcription factor c-Myc have been revealed as key regulators of *O*-GlcNAcylation at different stages of T cell development and differentiation (9). OGT and protein *O*-GlcNAc have also been identified as important for regulatory T (Treg) cell lineage stability and effector function (10). Additionally, the role of *O*-GlcNAcylation in some other classes of immune cells has recently started to be investigated, leading to interesting findings. For instance, the inhibition of OGA has been shown to promote apoptosis in activated B cells (11), whereas *O*-GlcNAc signalling has been found to inhibit proinflammatory macrophage activation (12). Over the last years, several excellent reviews have been published on the key roles of *O*-GlcNAc in the immune system, from reports focusing on immune cell function (13, 14) and activation (15), T-cell development (16), inflammation (17, 18) and infection (19) to more general overviews (20, 21). Considering the critical importance of *O*-GlcNAc in the immune system and the unclear molecular mechanisms underlying *O*-GlcNAcylation in immunity, further research efforts on the development of new chemical biology strategies will be important to crack the *O*-GlcNAc code in immune cells with potential therapeutic implications. In this minireview, we provide an overview of recent advances in deciphering the role of *O*-GlcNAcylation in the immune system using chemical tools.

## Chemical approaches for investigating *O*-GlcNAcylation in immune cells

Chemical biology approaches have yielded significant results in the interrogation and identification of the functions and consequences of *O*-GlcNAcylation in numerous biological settings and constitute promising tools to investigate the roles of *O*-GlcNAc in immune cells. Among the various strategies, here we focus mainly on synthetic OGT/OGA enzyme inhibitors exploited in the context of immunity as well as metabolic and chemoenzymatic labeling approaches applied to advance the *O*-GlcNAc field at the interface with immunology.

### Synthetic OGT and OGA inhibitors

In recent years, considerable efforts have been devoted to the development of potent OGT and OGA small-molecule inhibitors as valuable chemical tools to explore and control *O*-GlcNAcylation for therapeutic applications (**Table 1**) (22–24). OGT inhibitors have been developed using high throughput screening (HTS) of new compound libraries or by rational design of analogues of the enzyme substrate UDP-GlcNAc. Most OGA inhibitors have been designed by mimicking the transition state of species proposed in the substrate-assisted catalytic mechanism of OGA. Some of these compounds have been applied to interrogate *O*-GlcNAcylation in particular immune cells. In this section, we will highlight relevant studies using enzyme inhibitors categorized based on the immune cell type. Among all the immune cells, *O*-GlcNAcylation has been most widely investigated in T cells. These are one of the two main types of lymphocytes, with B cells representing the second major class, and are essential components of the immune system, being involved in determining the specificity of the immune response to antigens. In this context, an early study demonstrated that short-term activation of T cells results in rapidly decreased levels of *O*-GlcNAc modified proteins in the cytosol and, concomitantly, increased levels in the nucleus (25). However, the lack of advanced molecular tools hindered the rationalization of these observations, which was later possible thanks to the development of selective enzyme inhibitors that enabled the elucidation of key mechanistic insights. For instance, an OGA inhibitor (PUGNAc) was used in one such study where OGT was found to be required for lymphocyte activation (8). This work showed that PUGNAc treatment in a human lymphoma B-cell line (BJAB) increased *O*-GlcNAcylation of the transcription factors NF-κB (p65 subunit) and nuclear factor of activated T cells (NFAT), which in turn activated NFAT and promoted CD69 surface externalization, indicating a role for *O*-GlcNAcylation in early B cell activation. Further, inhibiting OGA with PUGNAc in peripheral blood mononuclear cells (PBMCs) led to activation of primary human B and T cells in a concentration-dependent manner, highlighting OGT as an essential signaling component downstream of the T cell and B cell receptors. Conversely, this study showed that OGT knockdown reduced NF-κB and NFAT activation with subsequent decrease in IL-2 secretion, consistent with the impairment of lymphocyte activation (8). In another example, the OGT inhibitor Ac_4_-5S-GlcNAc was used with stimulated primary T cells, leading to diminished production of IL-2 and supressed T cell proliferation (26). On the other hand, treatment with another potent OGA inhibitor, ThiamEt-G (35,000 more specific than PUGNAc) (27), had no influence on IL-2 levels, indicating that activity of OGT but not OGA is necessary for T cell effector function. While NF-κB and NFAT had earlier been shown to be key regulators for T or B cell activation, a more recent study has identified some other critical factors such as the PKC kinase, phosphorylate phospholipase C gamma 2 (PLC-γ2) and lymphocyte-specific protein-1 (Lsp1), which are also responsible for B cell activation and apoptosis (11). This work used Thiamet-G to analyze the functional interplay between protein *O*-GlcNAcylation and phosphorylation in stimulated mouse primary B cells. It was found that OGA inhibition induces apoptosis in activated B cells *via O*-GlcNAcylation of Lsp1 at S209, which in turn facilitates its phosphorylation at S243 by recruitment of the PKC-β1 kinase, ultimately leading to phosphorylation-mediated transduction of apoptotic signals.

**Table 1:** OGA and OGT inhibitors used in studies connecting *O*-GlcNAc and its role in immunity.

Compound name	Chemical structure	Inhibitory activity	Remarks
Main OGA inhibitors used in immune cells			
PUGNAc	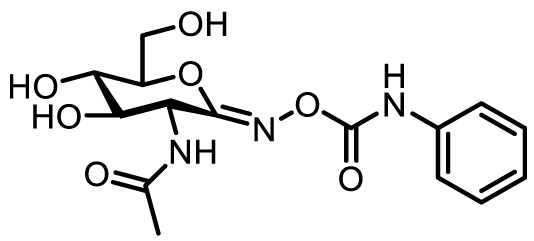	*K_i_ =* 46 nM	**+** Earliest and widely used **×** Selectivity issues versus other GH20 glycoside hydrolases. Care should be taken while interpreting data. • Applied to study B and T cells, (8, 28, 31) pancreatic β cells, (30) and macrophages (3, 32)
Thiamet-G	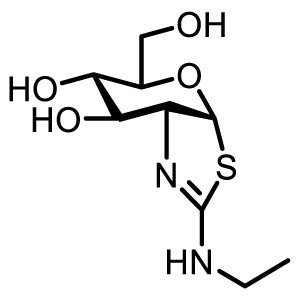	*K_i_* = 2.1 nM	**+** Highly potent and selective **+** Penetrates blood brain barrier • Applied to study T cells (29) including Tregs, (10) macrophages, (12, 33) NK cells (34)
GlcNAcstatin- G	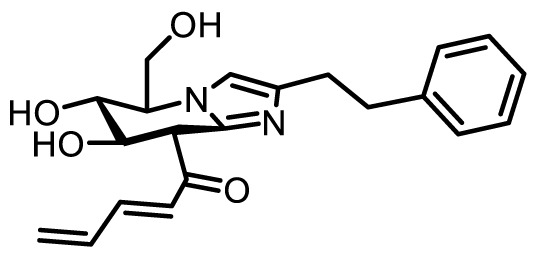	*K_i_* = 4.1 nM	**+** Very potent and cell penetrating **+** Extremely high selectivity (>900,000 fold) for hOGA over β-hexosaminidases • Applied to study T cells (9)
Main OGT inhibitors used in immune cells			
Ac_4_-5S-GlcNAc	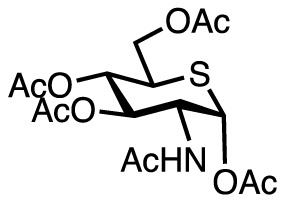	IC_50_ = 11 µM EC_50_ = 5 µM	**+** Potent inhibitor *in vitro* and in cell lines **+** Good cell permeability • Applied to study T cells (9, 26)
OSMI-1	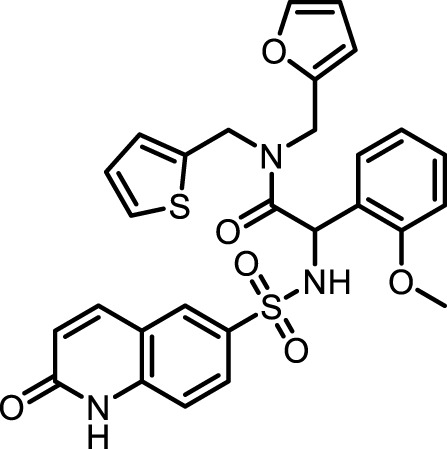	IC_50_ = 2.7 µM	+ Improved inhibitory potency × Less soluble in aqueous media, which hinders its inhibitory activity. • Applied to study NK cells (34) and DCs (35)

While *O*-GlcNAcylation is essential for vital cellular processes and normal T cell function, aberrant levels of this modification are linked to T-cell mediated inflammation and pathological conditions. For instance, the effect of *O*-GlcNAc under hyperglycemia in lymphocytes has been investigated (28). Using PUGNAc among other techniques, this study demonstrated that enhancement of *O*-GlcNAcylation of the NF-κB subunit c-Rel (at S350) increased DNA binding and subsequent transactivation, potentiating the expression of cytokine-encoding genes IL2, IFNG, and CSF2 after T cell receptor (TCR) costimulation. These results suggest a pathological role of *O*-GlcNAcylation in modulating c-Rel function by promoting T cell-mediated autoimmunity in diabetic conditions through increased production of T helper cell-1 type cytokines. Further research has shown the influence of elevated *O*-GlcNAc in CD4^+^ T cells in mediating a pro-inflammatory Th17 response in an obesity mouse model (29). Using Thiamet-G, the authors found a correlation between increased *O*-GlcNAcylation and higher pro-inflammatory IL-17A secretion by CD4^+^ T cells mediated by changes in intracellular lipid expression, pointing to a key role of *O*-GlcNAc in nutrient sensing and pathological inflammation by regulating Th17 cell function. Another study has shown that enhanced *O*-GlcNAcylation *via* PUGNAc treatment leads to glycosylation of the thioredoxin interacting protein (TxNIP) in pancreatic β cells, promoting its interaction with and activation of the inflammasome protein NLRP3 with induction of IL1β secretion (30). In an earlier work, treatment of human T lymphoblastic HPB-ALL cells with PUGNAc led to a considerable reduction in DNA fragmentation after induced apoptosis, which could be linked to *O*-GlcNAcylation of the DFF45 protein that inhibits the endonuclease DFF40, indicating a correlation between *O*-GlcNAc and DNA fragmentation during apoptosis in T cells (31). The use of OGT and OGA inhibitors has also been exploited in a study that identified a relationship between nutrient uptake, *O*-GlcNAcylation and c-Myc expression in the regulation of T cell function (9). The authors observed that TCR-activated T cells expressed lower levels of c-Myc in the presence of the OGT inhibitor Ac_4_-5S-GlcNAc, whereas the OGA inhibitor GlcNAcstatin-G caused the opposite effect, pointing to the importance of *O*-GlcNAcylation in regulating c-Myc expression in T cells. In addition to c-Myc, Notch and the TCR were also identified as key controllers of T cell protein *O*-GlcNAcylation *via* modulation of glucose and glutamine transport, emerging as metabolic checkpoints in T cell self-renewal, differentiation, and proliferation. More recently, *O*-GlcNAc has also been revealed as functionally important to control homeostasis and function in Treg cells by modifying the FOXP3 and STAT5 transcription factors (10). Inhibition of OGA by Thiamet-G enhanced FOXP3 expression and the number of CD4^+^ FOXP3^+^ Treg cells, indicating that *O*-GlcNAcylation stabilizes FOXP3 and Treg cell lineage. Moreover, Thiamet-G treatment increased the expression of STAT5-target genes and the suppressive activity of human Treg cells, revealing a key role for *O*-GlcNAc in maintaining the suppressive function of Tregs.

On the other hand, the application of enzyme inhibitors to explore *O*-GlcNAcylation in other immune cell types has been limited, with some recent examples having mainly focused on macrophages. In one study using PUGNAc, O-GlcNAcylation of STAT3, a well-known signal transducer of the cytokine-cytokine receptor signaling pathway, was shown to inhibit STAT3 phosphorylation and its transcriptional activity, with reduction of IL-10 production (32). *In vivo* mouse studies in a colitis-associated cancer model showed that defective anti-inflammatory STAT3–IL-10 activation promoted colonic inflammation and inflammation-driven tumors, suggesting that O-GlcNAcylation can affect inflammatory response via both pro- and anti-inflammatory signaling pathways. In another study, Thiamet-G was tested as a novel inflammation antagonist in a murine stroke model by evaluating inflammatory responses and microglia/macrophage polarization in mice (33). Thiamet-G treatment decreased pro-inflammatory cytokine expression, suppressing inflammation in this experimental model, and reduced the number of microglia/macrophages, inhibiting microglia activation. Moreover, Thiamet-G inhibited polarization to the M1 phenotype and NF-κB p65 signaling. These combined results highlight the therapeutic potential of Thiamet-G and of *O*-GlcNAcylation to ameliorate neurologic damage from stroke and promote functional repair. This protective role of *O*-GlcNAc has also been demonstrated previously in other experimental settings (e.g. in cardiac function after trauma-haemorrhage, and ischemia-reperfusion (I/R) injury) by using other less potent OGA inhibitors (e.g. PUGNAc, NAG-thiazoline) (36–38). Collectively, these studies showed that treatment with OGA inhibitors improves cardiac function and perfusion of critical organ systems, inhibited the infiltration of neutrophils and monocytes, and reduced inflammatory cytokines in plasma hindering acute inflammatory responses. A more recent work by Yang and co-workers using various OGA inhibitors (e.g. Thiamet-G, PUGNAc) has shown the effect of *O*-GlcNAcylation on suppressing macrophage inflammation *via* S6K1 glycosylation, inducing reduced expression of M1 polarization markers (12). This supports an immunosuppressive role of *O*-GlcNAc in macrophage activation, which contributes to metabolic homeostasis at the early stage of obesity, thus functioning as a homeostatic regulator integrating immunity and metabolism. To date, efforts focused on deciphering the role of *O*-GlcNAc in other immune cells such as NK cells or dendritic cells have been scarce. A very recent study by Parameswaran and co-workers has probed the effect of *O*-GlcNAcylation on modulating NK cell cytotoxic function. The authors showed that OSMI-1, a potent OGT inhibitor, decreases the cytotoxic activity of NK cells against various cancer cells (associated with reduced expression of NKG2A/D surface receptors, TNF-α and IFN-γ cytokines, and cytotoxic mediators), whereas Thiamet-G treatment did not affect NK cell cytotoxicity (34). Another recent work has explored the role of *O*-GlcNAc in dendritic cells (DC), revealing that OGT inhibition by OSMI-1 altered several signaling pathways (MEK/ERK and mTOR/AKT) as well as cytokine and surface marker expression. Altogether, OSMI-1 treatment impaired the differentiation and maturation process of monocytes (mo) into DCs, hampered their endocytic capacity, and increased their ability to promote T cell proliferation (35). Taken together, these studies combining chemical inhibition with genetic strategies to modulate *O*-GlcNAcylation provide important insights into the role of *O*-GlcNAc in immunity with potential therapeutic implications, while also highlighting the need for investigating its specific effects for each immune cell type. In conclusion, a summary of the main effects of pharmacological OGA and OGT inhibition in key signaling pathways in immune cells is provided in **Table 2** below.

**Table 2:** Effect of OGA and OGT pharmacological inhibition in immune cell signaling pathways.

*O*-GlcNAc levels (inhibitor)	Immune cell type	OGA/OGT inhibition effects in immune cell signaling pathways
High (PUGNAc)	B and T cells	• Increased NF-κB (p65 subunit) and NFAT *O*-GlcNAcylation; NFAT activation ➔ Primary human B and T cell activation (8).
High (Thiamet-G)	B cells	• Elevated Lsp1 *O*-GlcNAcylation; increased recruitment of PKC kinase ➔ Enhanced B-cell activation and apoptosis (11).
High (PUGNAc)	T cells (hyperglycemia)	• Increased NF-κB (c-Rel subunit) *O*-GlcNAcylation; induced c-Rel transactivation and Th1-type cytokine production ➔ Enhanced T cell-mediated immune responses and autoimmunity (28)
High (Thiamet-G)	CD4^+^ T cells (obesity model)	• Altered intracellular lipidome; increased pro-inflammatory IL-17A cytokine production ➔ Enhanced Th17 cell function (29)
High (PUGNAc)	Pancreatic β-cells(diabetic)	• Elevated TxNIP *O*-GlcNAcylation; increased TxNIP-NLRP3 interaction ➔ NLRP3 inflammasome pathway activation (30)
High (PUGNAc)	T lymphoma cells	• Increased DFF45 *O*-GlcNAcylation; inhibition of DFF40 endonuclease ➔ Reduced DNA fragmentation during apoptosis (31)
Low (Ac_4_-5SGlcNAc)	Activated T cells	• Decreased c-Myc *O*-GlcNAcylation and expression ➔ failed T cell clonal expansion, self-renewal, and malignant transformation (9)
High (GlcNAcstatin-G)	Activated T cells	• Increased c-Myc *O*-GlcNAcylation and expression ➔ induced T cell differentiation, proliferation, and tumor formation (9)
High (Thiamet-G)	Treg cells	• Elevated FOXP3 and STAT5 *O*-GlcNAcylation; increased FOXP3 stabilization and STAT5 activation ➔ Enhanced Treg cell lineage stability and suppressive effector function (10)
High (PUGNAc)	Colon macrophages	• Elevated STAT3 *O*-GlcNAcylation, decreased STAT3 phosphorylation and activation; reduced IL-10 production ➔ ➔ Exacerbated intestinal inflammation driving tumorigenesis (32)
High (Thiamet-G)	Macrophages	• Decreased pro-inflammatory cytokine expression; enhanced M2 and inhibited M1 polarization marker expression; suppressed NF-κB (p65) (33) and S6K1 (12) signaling ➔ Inhibited microglia activation and S6K1-mediated macrophage inflammation (12, 33)
Low (OSMI-1)	NK cells	• Decreased TNF-α/IFN-γ and cytotoxic mediator expression ➔ ➔ Reduced NK cell cytotoxic function (34)
Low (OSMI-1)	moDCs	• Decreased MEK/ERK & mTOR/AKT phosphorylation; altered DC surface marker/cytokine expression ➔ impaired moDC maturation and endocytic capacity, and increased T cell stimulation ability (35)

The arrow means the ultimate consequence/impact in pathways/immunity of the preceding effects caused by OGA/OGT inhibition (i.e. "ultimately leading to")

### Metabolic and chemoenzymatic *O*-GlcNAc labeling

**Figure 1:**
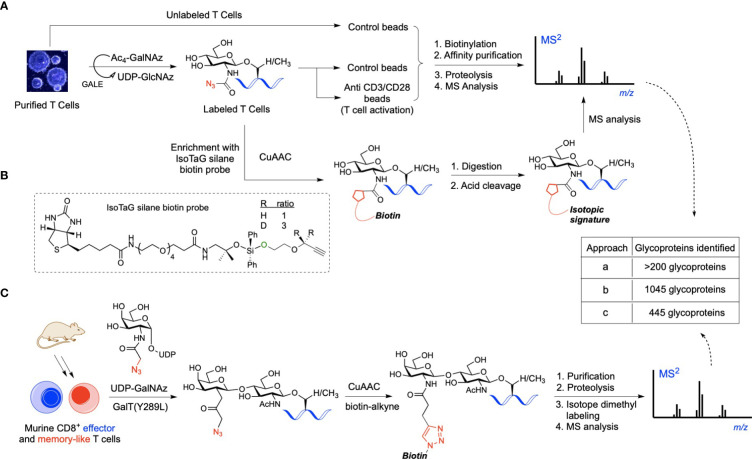
**(A)** Workflow of *O*-GlcNAc metabolic labeling for the identification of *O*-GlcNAc proteins in activated human T cells. Cells were incubated with Ac_4_GalNAz and stimulated with control beads or anti-CD3/CD28 beads for T cell activation. Azido-labeled proteins were biotinylated for affinity enrichment using streptavidin beads, then purified and digested for MS analysis, identifying relevant *O*-GlcNAc proteins. **(B)** IsoTaG labeling strategy involving click chemistry-based tagging of the azido-labeled proteins with the IsoTaG silane probe incorporating an acid-cleavable biotin and an isotopic label with a terminal alkyne. Biotinylated glycoproteins were affinity purified, digested, and retrieved *via* acid-mediated cleavage for subsequent LC-MS/MS sequencing. **(C)** Chemoenzymatic profiling of *O*-GlcNAc proteins in mouse CD8^+^ effector- and memory-like T cells using UDP-GalNAz as the unnatural nucleotide donor. Probing of the GalNAzylated *O*-GlcNAc proteins with a biotin tag *via* click-chemistry, followed by affinity purification, digestion, and isotope dimethyl labeling yielded *O*-GlcNAc glycopeptides for MS analysis, enabling the identification of unique *O*-GlcNAc-enriched proteins important in CD8^+^ T cell function.

Until recently, there were essentially no reports on the use of advanced chemical approaches for the identification of *O*-GlcNAcylated proteins in immune cells. In 2016, Lund et al. utilized metabolic glycan labeling with tetraacetylated *N*-azidoacetylgalactosamine (Ac_4_GalNAz) as an unnatural substrate reporter, identifying >200 *O*-GlcNAc proteins in *in vitro* activated human T cells (**Figure 1A**) (26). In this chemical biology strategy, originally developed by Bertozzi and co-workers, the synthetic Ac_4_GalNAz monosaccharide is fed to cells and then metabolized intracellularly to UDP-GlcNAz *via* the UDP-galactose 4-epimerase (GALE) pathway, serving as a nucleotide sugar donor for OGT to yield chemically tagged *O*-GlcNAzylated proteins (39). These azido-labeled glycoproteins can be captured with a biotin probe using bioorthogonal chemistry, enabling enrichment and profiling of *O*-GlcNAc-modified proteins in relevant cellular systems. In this early study, T cell activation was confirmed to result in elevated *O*-GlcNAcylation with many of the identified glycoproteins having a functional relationship with RNA metabolism (26), albeit details of glycosylation points and functionally important modification sites during activation could not be elucidated. In a subsequent study, Woo and co-workers developed further this approach by implementing the so-called IsoTaG (Isotope-Targeted Glycoproteomics) technology to map and quantify *O*-GlcNAc sites in resting versus activated human T cells (**Figure 1B**) (40). Thus, the metabolically labeled *O*-GlcNAcylated proteins incorporating the azido chemical handle were tagged with a cleavable, isotopically encoded biotin probe *via* click chemistry for subsequent affinity enrichment. The digested glycopeptides were recovered by cleaving the IsoTaG biotin moiety and analyzed by mass spectrometry, enabling the identification of over 2,000 unique *O*-GlcNAcylated glycopeptides from >1,000 glycoproteins in human T cells and confirming the functional role of *O*-GlcNAc in T cell activation. However, Ac_4_GalNAz has been found to also result in non-specific incorporation into other forms of glycoconjugates and competes with the native UDP-GlcNAc pool, leading to an inaccurate characterization of *O*-GlcNAc abundance. To avoid nonspecific labeling and enable a more comprehensive profiling, another alternative approach was developed by the Wu laboratory combing chemoenzymatic glycan labeling and proteomic analysis to compare protein *O*-GlcNAcylation in *in vitro* differentiated murine effector- and memory-like CD8^+^ T cells (**Figure 1C**) (41). Their approach builds upon the methodology originally developed by the Hsieh-Wilson group (42), and exploits the β1,4-galactosyltransferase mutant (GalT1 Y289L) to incorporate a GalNAz reporter to *O*-GlcNAc modified proteins in CD8^+^ T cells before and after activation. Chemoselective installation of a biotin-alkyne probe to the GalNAz modified *O*-GlcNAcylated proteins *via* click chemistry, followed by affinity enrichment and on-bead digestion enabled the identification of 445 unique proteins, many of them phosphorylated and not previously reported. Some of the characterized glycoproteins were found to be important for mRNA processing in the case of memory T cells and involved in transcription and translation for effector T cells, confirming the significance of *O*-GlcNAcylation in T cell biology. Despite these important examples, improved and modern chemical approaches are needed to complement traditional genetic strategies for deciphering, at the molecular level, the largely unclear role of *O*-GlcNAc in the immune system, especially in other immune cell types beyond T cells. In **Table 3**, we outline the functional relationships and potential pathways associated with the identified *O*-GlcNAcylated proteins during T cell activation in the above glycoproteomics experiments.

**Table 3:** Functional links between the identified *O*-GlcNAc proteins in T cells and potential associated pathways.

Immune cell type	Identified glycoproteins data and associated pathways/functions
Activated human T Cells	• 214 *O*-GlcNAcylated proteins identified • Functionally related to RNA transcription and metabolism (26)
Activated human T Cells	• >45% *O*-GlcNAc sites lie near phosphorylation sites, supporting PTM crosstalk • Increased c-JUN and JUNB *O*-GlcNAcylation and expression levels (40) • Functionally linked to transcriptional regulation and TCR signaling (40)
Murine effector- and memory-like CD8^+^ T cells	• >70% glycoproteins known to be phosphorylated • Several *O*-GlcNAcylated protein subsets enriched for each CD8^+^ T cell type • Involved in transcription and translation for effector T cells (41) • Involved in mRNA processing for memory T cells (41)

## Discussion and conclusion

With the advancement of new chemical tools, considerable progress has been made in the field of *O*-GlcNAc at the interface with immunology. Over the last decade, several OGT and OGA inhibitors with high potency, specificity, and encouraging cell permeability have been developed and successfully applied to study *O*-GlcNAcylation in immunity. Yet, there is a clear need for improved enzyme inhibitors for *in vivo* studies as well as substrate-specific inhibitors that do not interfere with the global *O*-GlcNAc levels required for optimal immune cell function. Other chemical approaches such as chemoenzymatic and metabolic labeling strategies in combination with advanced glycoproteomics techniques have been exploited as useful tools to profiling *O*-GlcNAc proteins in immune cells, particularly T cells. Nonetheless, additional studies are warranted for the investigation of *O*-GlcNAcylated proteins in other immune cells applying these methodologies as well as for the development of further optimized chemical/biological tools that can facilitate a more efficient probing of *O*-GlcNAc in cells under near-native conditions. Thus, new improved technologies for tracking *O*-GlcNAc *in vivo* will make possible to monitor and manipulate *O*-GlcNAcylation in a specific protein and cellular localization, contributing to the elucidation of the precise roles of *O*-GlcNAc in specific immune cell types. In 2017, Van Aalten and co-workers developed a genetic recoding approach exploiting Ser/Thr to Cys mutagenesis and the promiscuity of OGT to genetically introduce a non-hydrolyzable *S*-GlcNAc surrogate. Unlike OGA inhibition, this strategy enabled precise functional dissection of site-specific *O*-GlcNAcylation *in vivo* without affecting the entire *O*-GlcNAc proteome (43). Notably, this technique can be combined with the CRISPR-Cas9 genome editing technology for further application in mammalian systems, emerging as a potentially powerful tool for interrogating *O*-GlcNAc in live human immune cells in a site-specific manner. On the other hand, chemical synthetic approaches based on protein ligation methods provide a useful alternative for the preparation of homogenous *O*-GlcNAcylated proteins for further biochemical studies (24), albeit they have not yet been applied to investigate functionally important *O*-GlcNAc glycoprotein targets in immunity. In summary, it will most likely be a combination of chemical and genetic strategies with their respective strengths and limitations that will provide the community with improved tools to study and decipher the role of the *O*-GlcNAc modification in the immune system with potential for immunotherapeutic development.

## Author contributions

AS conducted the literature search, wrote the first draft, and made the figures. AF-T gave direction for sections, designed the figures, revised and wrote the final version. All authors contributed to the article and approved the submitted version.
